# Combined surgical and negative pressure therapy to treat multiple enterocutaneous fistulas and abdominal abscesses: A case report

**DOI:** 10.1016/j.amsu.2020.06.037

**Published:** 2020-07-15

**Authors:** Gaetano Luglio, Alfonso Amendola, Gianluca Pagano, Francesca Paola Tropeano, Chiara Errico, Enrica Esposito, Giuseppe Palomba, Paola Dinuzzi, Giuseppe De Simone, Giovanni Domenico De Palma

**Affiliations:** Department of Public Health. University of Naples "Federico II", Naples, Italy

**Keywords:** Negative pressure therapy, Enterocutaneous fistulas, VAC therapy, abdominal wall closure

## Abstract

**Introduction:**

We report the case of a successful management with combined aggressive surgery and negative pressure therapy, to treat a severely ill-septic patient, affected by multiple chronic enterocutaneous fistulas.

**Presentation of case:**

A 26-year-old female patient presented with multiple pelvic and intra-abdominal abscesses, enterocutaneous fistulas and central venous catheter-related bacteraemia in extremely poor general conditions.

The patient underwent both an abdominal CT which showed multiple digestive loops stuck and apparently fistulised and an abdominal-pelvic MRI, confirming the CT findings, and demonstrating a third fistula involving the Pouch and responsible for a pelvic and retroperitoneal chronic abscess.

Given the patient's septic condition, despite several attempts of conservative therapies, an aggressive surgical approach was adopted.

After temporary abdominal wall closure, the patient underwent Vacuum Assisted Closure therapy in order to close the abdominal wall and drain the residual abscess. The patient was discharged at the 35th post-operative day in good general conditions.

**Discussion:**

This case is about a complex, long-lasting clinical scenario, progressively leading a young woman to death despite several attempts of conservative therapy, sometimes allowed to treat enterocutaneous fistulas. The use of negative pressure therapy to manage open abdomen is still controversial. Patients affected by enterocutaneous fistulas are in need of adequate nutritional support due to their hypercatabolic state, secondary both to the fluid loss and the concomitant inflammatory status.

**Conclusion:**

When conservative management fails and the patient shows septic complications, a multidisciplinary aggressive approach, including surgery, negative-pressure therapy and hyperbaric oxygen therapy is required to treat this life-threatening condition.

## Highlights

• Using negative pressure therapy to deal with difficult abdominal wall closure.
• Multidisciplinary team work is essential to optimize patients' surgical outcomes.
• Highly-demolitive surgery can benefit from the use of negative pressure therapy.

## 1 Introduction

This case report has been reported in line with the SCARE criteria [1].

Entero-cutaneous fistulas (ECFs) are abnormal connections between the lumen of the intestine and the cutaneous surface. They can be classified according to several aspects such as their daily-output (High>500 cc/day, Moderate 200–500 cc/day, Low <200 cc/day), their location (proximal or distal), their aetiology (iatrogenic or spontaneous), and their numerosity (single, multiple).

The global incidence of ECFs is still unknown but data from retrospective studies state that in the 25% of cases they are secondary to other clinical conditions (Inflammatory Bowel Disease, diverticulitis, traumas, neoplasms, radiotherapy) while in the 75% ECFs arise from a post-operative anastomotic leak [2].

The use of negative pressure (VAC Therapy) is still controversial as there is not clear evidence from literature: some studies report an increase in the incidence of ECFs while some others state the opposite [3,4].

In the present case report, we will discuss about the successful management with combined aggressive surgery and negative pressure therapy (NPT), to treat a severely ill-septic patient, affected by multiple chronic enterocutaneous fistulas.

## 2 Presentation of case

A 26-year-old female patient was admitted at the Infectious Diseases Department at Federico II University Hospital, because of multiple pelvic and intra-abdominal abscesses, enterocutaneous fistulas and central venous catheter-related bacteraemia. Three years before the admission, the patient underwent proctocolectomy with ileal pouch for Familial Adenomatous Polyposis type 1 (FAP1) [5].

At the time of the admission, the patient was in extremely poor general conditions (Table 1), her nutritional status was severely compromised as she had been managed with total parenteral nutrition (TPN) for two years with a body mass index (BMI) of 16,4 kg/m^2^.

**Table 1:** Patient's pre-operative and post-operative characteristics.

	Pre-operative	Post-operative month n.6
**Weight (kg)**	43	76.1
**Height (cm)**	162	162
**BMI (kg/m**^**2**^**)**	16.4	29.1
**Evacuations (n/day)**	4	3 (stoma bags)

**Fig. 1:**
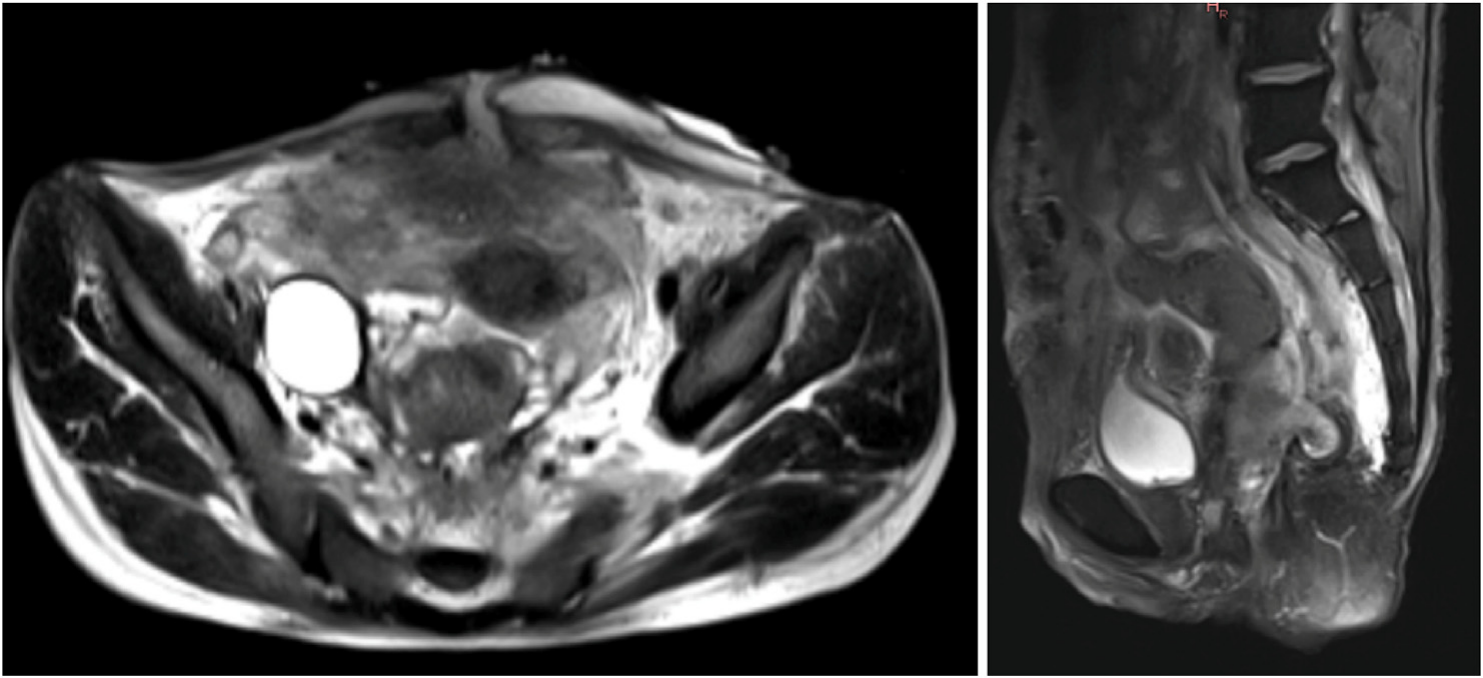
Abdominal MRI - A poorly-dissociable conglomerate of ileal loops was also described, specially involving the pelvic and terminal ileum, with widespread thickening and enhancement of the mesentery, due to phlogosis, and several entero-enteric/entero-mesenteric fistulas.

During hospitalization, several diagnostic tests were performed including B-*d*-glucan, Rectal Buffer, urine culture, haemoculture. Furthermore, the patient underwent abdominal CT (with/without contrast) showing a diffuse inhomogeneity of the adipose peritoneal cellular layer in the pelvic cavity, with multiple digestive loops stuck and apparently fistulised. Then, an abdominal-pelvic MRI was performed, confirming the CT findings, and demonstrating a third fistula involving the Pouch and responsible for a pelvic (pre-sacral) and retroperitoneal chronic abscess. (Fig. 1). Particularly, there was a diffuse and intense transmural enhancement of the anastomosis wall with the presence of two fistulous abscesses: the first one posterior towards the left iliac-obturator space; the second showed a horseshoe-shaped course, close to the pouch walls. The presence of two entero-cutaneous fistulas of the hypogastric region was also reported.

Given the patient's septic condition, with highly increased markers of systemic inflammation, despite several attempts of conservative therapies, an aggressive surgical approach was proposed [6].

The patient underwent xipho-pubic laparotomy with ileal resection, demolition of the pouch and terminal ileostomy construction. The surgical équipe was made of consultant colorectal surgeons and colorectal surgery residents.

**Fig. 2:**
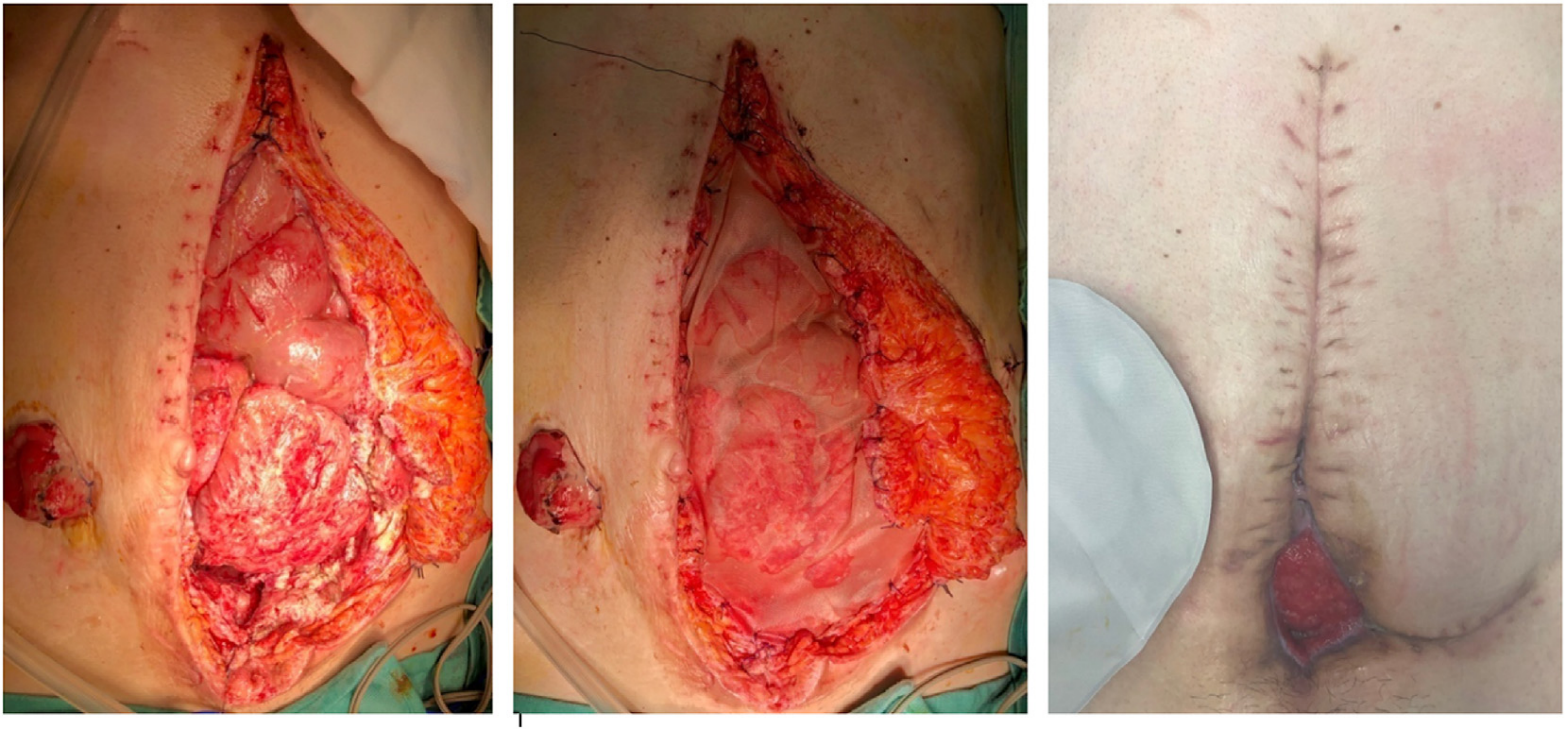
Abdominal wall during surgery and at 6th post-operative month.

After midline re-laparotomy, an impenetrable abdomen with very strong adhesions was found. After careful adhesions division, a multi-fistulising abscessual block was found, starting from the root of the mesentery and spreading into the retro-peritoneal space, then going down to the pelvis, in communication with a posterior dehiscence of the anal pouch anastomosis. The ileum involved in the enterocutaneous fistulas was resected. A terminal high ileostomy was fashioned. The pouch was also removed, requiring an abdominoperineal amputation. Pelvic packing was required for 48 hours, as a complete haemostasis was not achievable due to the inflammation of the abscess wall. A complete abdominal wall closure was not feasible, therefore a negative pressure therapy device, with an open abdomen kit, was required (Fig. 2). On third post-operative day (POD), we removed both the mesh and abdominal-pelvic gauzes. Abdominal wall closure was achieved through a VICRYL™ (polyglactin 910) mesh. After consecutive abdominal cavity washes and haemostasis check, we placed a VAC therapy device. At the end of surgery, the length of the remaining bowel was 1.5 m.

During her post-operative course, the patient was administered with antibiotics, antifungin, albumin and anticoagulant drugs. Her phlogosis indexes (CRP and PCT) were daily tested whilst there was a weekly evaluation of B-*d*-glucan, chemical physical urine status, urineculture and serial blood cultures exams both from central venous catheter (CVC) and peripheral catheter (PVC) in case of fever.

The VAC therapy device was replaced every 72 hours and completely removed after re-granulation of the anterior abdominal wall on 25th POD. The patient underwent hyperbaric oxygen therapy (HBOT) for 15 days. She was finally discharged on 35th POD with trophic, active and well-functioning stoma with haematochemical values within the normal range, in definitely improved general conditions. As far as the nutritional status in concerned, we experienced a significant improvement. In addition to the BMI, the laboratory tests improved too: preoperative albumin value was 1.8 g/dL versus 4 g/dL postoperatively (n.v. 3.5–5.2 g/dL) while the preoperative serum total protein value of 4 g/dL increased to 6 g/dL (n.v. 6.4–8.3 g/dL) at 12th POD. Immediately after surgery, the patient received TPN which was accompanied by oral nutrition from the 7th POD. Exclusive oral nutritional therapy was administered since the 15th POD. According to both nutritionists’ and our opinion, the rapid BMI increase was due to both to the resolution of her chronic catabolic inflammatory status and the nutritional therapy provided with high calorie and protein intake.

The patient has been followed-up at our general surgery ambulatory for post-operative care and is currently in very good general conditions. She is monthly followed-up by our nutritionists team and is getting continuous improvement (Table 1). Six months after the surgical intervention, the patient was in excellent clinical conditions with negative phlogistic indexes and weight increase, having reached 76,1kg (BMI 29,04 kg/m^2^) with a remarkable improvement of her performance status.

## 3 Discussion

Entero-cutaneous fistulas (ECFs) are such a challenging surgical scenario and still represent an important cause of post-operative morbidity/mortality: this is the reason why they are a highly-debated topic in General Surgery.

As previously stated, the use of NPT is still controversial as there is not clear evidence from literature: some studies report an increase in the incidence of ECFs while some others state the opposite [3,4].

Literature studies regarding the use of NPT to manage open abdomen present consistent limitations due to enrolled-patients’ heterogeneity [7,8], to the variety of the evaluated technologies (craft versus commercially available systems) and to the different pressure values and modalities (continuous or intermittent).

The National Institute for Health and Care Excellence (NICE) recommends [9] the dressing-up to be performed by professionals with an expertise in order both to recognise and avoid early complications such as intestinal fistulas and perforation, bleeding, infections, hernias, incisional hernias and pain. In any case, the 2013 NICE [9] indications state to adopt the NPT for a short period of time. In addition, the device can be adopted as a first-line strategy to obtain an abdominal decompression in case of anasarca and/or abdominal compartment syndrome.

Patients affected by ECFs are in need of adequate nutritional support due to their hypercatabolic state, secondary both to the fluid loss and the concomitant inflammatory status. Therefore, it is mandatory to correct electrolyte disorders, daily evaluation of the acid-base balance and the fluids input/output.

Parenteral nutrition (PN) has been the standard therapy for many years even though, nowadays, several studies demonstrated that enteral nutrition (EN) can be approachable and effective in reducing infectious complications [10].

Good prognostic factors of possible conservative fistula resolution include good nutritional status, serum albumin > 3g/dL, diverticular disease-related fistula, low fistula output, single fistula, intestinal continuity, whereas negative prognostic factors are generally poor nutritional status, low serum albumin/ferritin levels, intestinal abnormality >1cm, high fistula output, previous radiotherapy and surgical interventions [11].

Spontaneous fistula healing is very tough to be obtained, even more when dealing with deep fistulas and patients with underlying diseases.

In particular, once an ECF is diagnosed, it should be necessary to explore the abdominal cavity to evaluate the presence of misrecognised fistulas, underlying gastrointestinal diseases, foreign bodies, neoplasms and intestinal obstruction. If one of these conditions is found, the initial management should include its resolution, dealing with the fistula afterwards [12].

When dealing with small, superficial, distal and low-output fistulas, a primary closure could be attempted with sutures and several types of glue (fibrin glue and cyanoacrylate glue) [13].

In most cases, surgeons tend to perform a two-stage procedure: ECF removal at first and secondly the closing of the abdominal wall, once a proper physiologic homeostasis is obtained (8–12 months after surgery).

However, in our case, given both local and general patient's clinical conditions, also including multiple abdominal abscesses and pouch leak, we decided to proceed with a resolution of the ECFs as first instance. We performed a challenging adhesiolysis of the small intestine, the removal of the pre-existing fistulising pouch, the 50cm-long ileal resection and the construction of a terminal high ileostomy.

Then, the residual abdominal abscesses were drained thanks to a negative-pressure device [14] and the abdominal wall was closed only with a VICRYL™ mesh (polyglactin 910) and left for secondary healing.

The risk for intestinal failure (IF) and short-bowel is another issue to take into account when dealing with ECFs.

IF is characterised by the inability of the gut in absorbing macronutrients (carbohydrates, proteins and fats), micronutrients (vitamins, electrolytes and minerals) and water with the mandatory parenteral integration of them [15]. It can be either congenital or acquired, acute or chronic, auto-limited or not.

It can be classified as [16]:

- Type 1: secondary to an acute condition and, usually, auto-limiting (ex. post-operative ileus)
- Type 2: due to an abdominal surgical intervention in critic patients with multiple fistulas. It requires a multidisciplinary approach along with weekly or monthly-lasting parenteral nutrition.
- Type 3: the chronic one, in which patients are in need of a long-lasting parenteral nutrition. It can be reversible [17]. Also, it can be either the evolution of a Type 2 IF or the result of gastrointestinal diseases which require multiple intestinal resections.

IF average survival is high when it is due to a benign condition, reaching the 80% in adults and 90% in children [18]. Unfortunately, these percentage collapse in presence of comorbidities and septic scenarios.

## 4 Conclusion

ECFs fistulas are a challenging and high risk situation. When conservative management fails and the patient shows septic symptoms, a multidisciplinary aggressive approach, including surgery, patient optimization, negative-pressure therapy and HBOT is required to treat this life-threatening condition.

## Consent of patient

Written informed consent was obtained from the patient for publication of this case report and accompanying images. A copy of the written consent is available for review by the Editor-in-Chief of this journal on request.

## Provenance and peer review

Not commissioned, externally peer reviewed.

## Funding

None.

## Ethical approval

This paper was not a research study, so ethical approval not required.

## Registration

Name of the registry:

Unique Identifying number or registration ID:

Hyperlink to your specific registration (must be publicly accessible and will be checked):

## Guarantor

Prof. Gaetano Luglio is the Guarantor of this case report.

## CRediT authorship contribution statement

**Gaetano Luglio:** Conceptualization, Writing - original draft. **Alfonso Amendola:** Writing - original draft. **Gianluca Pagano:** Writing - original draft. **Francesca Paola Tropeano:** Writing - original draft. **Chiara Errico:** Data curation, Writing - original draft. **Enrica Esposito:** Data curation, Writing - original draft. **Giuseppe Palomba:** Writing - original draft. **Paola Dinuzzi:** Writing - original draft. **Giuseppe De Simone:** Writing - original draft. **Giovanni Domenico De Palma:** Conceptualization, Writing - original draft.

## Declaration of competing interest

All authors disclose any financial and personal conflict of interest.
